# Dental Convention of Northern Ohio

**Published:** 1857-12

**Authors:** 


### Proceedings of Societies.

### DENTAL CONVENTION OF NORTHERN OHIO.

According to previous announcement by circular, the Dental Profession of Northern Ohio, assembled in convention, in Tremont Hall, Cleveland, Ohio, on Tuesday, November 3d, 1857.

Members present: F. S. Slosson, B. Strickland, E. Merritt, L. C. Ingersoll, W. H. Atkinson, B. F. Robinson, T. M'Cune, J. A. Robinson, Joseph Willson, Wm. Fiske, N. H. Ambler,

Henry Slosson, J. E. Robinson, J. G. Moore, E. G. Burger, J. G. Rockwell, W. P. Horton, Cleveland; D. L. Norcross, Elyria; J. E. Atkinson, Millersburg; W. B. Ingersoll, Oberlin; Alfred Terry, Norwalk; C. H. Harroun, Toledo; Wm. E. Dunn, Delaware; C. F. Ingersoll, Huron; S. P. Huntington, Painesville; L. T. Fox, Madison; D. F. Knapp, Berea; C. S. Pleasants, Painesville; W. F. Robinson, Youngstown; B. T. Spelman, Ravenna; E. Chidester, Massillon; C. P. Bailey, Cuyahoga Falls; A. A. Harris, Ravenna; A. Barrett, Jefferson; J. G. Willis, Ravenna; M. J. Dickerson, Cleveland; Geo. Watt, Cincinnati.

The Convention was called to order by Dr. B. Strickland, and, on motion, Dr. F. S. Slosson was elected temporary chairman, and Dr. L. C. Ingersoll, Secretary. The call for the convention was read by the Secretary.

A committee of five was appointed to report a plan of organization and order of business. The committee consisted of Drs. Thomas McCune, C. S. Pleasants, W. B. Ingersoll, C. H. Harroun and Alfred Terry.

In the absence of the committee, Dr. J. A. Robinson, in behalf of those who called the Convention, gave a brief and eloquent salutatory address, which elicited hearty applause. Drs. Willson moved that Dr. R. be requested to give a synopsis of his remarks, for publication, with the proceedings. The motion carried.

While the committee was absent, Dr. J. A. Robinson arose and said:

Gentlemen of the Convention:—Perhaps it will not be assuming in me, or consuming the time of the Convention unprofitably, if I state, now, the object of this association, and, in a brief manner, express to you the great gratification it is to the dentists of Cleveland, that so many members of our profession have responded to our call.

Most of us have undoubtedly felt the need of an association of this kind, for a long time; and more especially for the last few years, during which we have had the privilege of reading the reports of the various dental conventions held in different parts of our country.

The advantages of associated labor in every department o mechanical industry, of the arts and of the sciences, have been fully established. We can see them in every manufacturing establishment, and in every institution of learning; and while we behold societies formed in every county in the State, for the exhibition and improvement of agricultural products and implements, and associations in every village for the amelioration of the condition of humanity, shall we any longer neglect to associate, for the elevation of our own profession? a profession that is destined ere long to rank among the most useful and honorable in the world.

Gentlemen, we meet for the elevation and the improvement of ourselves. Our patrons demand it. It is imperatively incumbent upon us, not only to know, but thoroughly to understand the most approved method of treating the deciduous teeth of the children placed under our charge; of regulating the teeth of the second dentition; and of so treating diseased teeth, that they will be saved without cavil or dispute.

I trust it will not be the misfortune of any members of this Convention, to appear egotistic in expressing themselves, or to expect to make themselves appear great, by endeavoring to make others appear small. Let there be a cordial manifestation of civility and courtesy in all our communications, that each one of us shall feel that he has been benefitted by the suggestions of every other.

And while we would have a degree of enthusiasm, such as every good dentist must feel when he looks upon a tooth well plugged, and feels that it is redeemed and saved, I trust we shall have nothing to interrupt the harmony of the occasion. Let us "keep the unity of the spirit in the bonds of peace."

There must, necessarily, be differences of opinion among us, both in regard to the various methods of performing certain operations, and in regard to professional fees. But I can assure you, gentlemen, that the more thoroughly a man is able to perform a difficult operation, either in surgical or in mechanical dentistry, the more perfectly will he be able to appreciate the worth of such an operation, and the more surely to find himself in a condition to command, for the same, a just remuneration.

It will not be improper in this Convention, to discuss the comparative values of the different kinds of artificial dentures, for there is probably no other section of the country in which they have been more thoroughly tested than Northern Ohio; and no other in which their true merits, both relative and absolute, are more certain to be ultimately appreciated.

Whatever we undertake, let us do it in the most thorough manner, making it the *very best of its kind.* Let us "mind our own business." If we do this, we shall hear less about the comparative worth of the different kinds of artificial teeth. For it is the working of a set of teeth, that makes it valuable to the wearer; and even that depends somewhat upon the *genius* of the wearer. It is an incalculable benefit to supply a perfect set of artificial teeth, as thousands can testify. But, while we admit that fact, we only prove more strongly the necessity of preserving the natural organs, which are as much superior to the artificial, as Nature is superior to Art.

Another object of this Convention is to elevate the standard of education.

Many of us who have been struggling up through a long labyrinth of adverse circumstances, for the last twenty years, can scarcely tell by what process we have been made what we are; but we are sure that it has been one severe and continued effort; and that too, sometimes, almost in the dark.

But circumstances have changed. That which was once so obscure and difficult, is now comparatively plain and easy.

The younger members do not have to travel over all the ground we have trod. They begin at our stand point, and take up the work where we leave off.

Dentistry is made up of little things, and it is the little things that make a good dentist. The ability to make a good instrument, decides the ability to plug a tooth well; and the care and skill in taking an impression and making a swage, decides the fitting of a plate.

It is no wonder that a man who learns his business in three weeks, and plugs a tooth that will last only three weeks, can not appreciate good dentristy, and thinks a dentist who gets a higher price than himself charges too much, and is guilty of extortion. For it is only by comparison that we discern between the good and the bad, and a dentist must be conversant with the conditions of the good, to discover the defects of the bad.

The object of this Convention will have been accomplished, and its benefits fully realized, if, by its proceedings, the strong shall be induced to assist the weak,—to hold up the hands of the latter until they likewise shall have become strong; for then, and not till then, will our profession be appreciated by the community at large, and the dentists themselves be remunerated for their services.

Gentlemen, in behalf of the dentists of Cleveland, I bid you welcome to this our first great *feast.* May you all partake and be satisfied.

The committee being still absent, Dr. W. H. Atkinson made some interesting remarks in regard to the inception of the call for this Convention, and the objects to be accomplished by properly conducted dental associations. He alluded to the resolution of the American Dental Convention, recommending the formation of local societies, and said that the call for this meeting was a direct result of that recommendation. For himself, he felt like *acting* on that resolution, as soon as proposed, at the Boston meeting; and, from the numbers already present, he inferred that others possessed the same feelings. By meeting in convention, face to face, professional jealousies are dispelled, and we feel like brethren. And this is not all. Our professional energies are waked up, and our minds are stored anew by exchange of ideas, till each one feels able, and determined to accomplish more in his profession than ever before.

It being the hour of noon, the Convention adjourned till 2 o'clock, P. M.

FIRST DAY—AFTERNOON SESSION.

The convention met at the appointed hour. Dr. McCune, Chairman of the committee on organization and business, presented the following report:

*Report of the Committee.*

Mr. President and Gentlemen :—In discharging the duties imposed on us, we would respectfully present the following form of organization and order of business:

ORGANIZATION. *Name.* This association shall be called the DENTAL CONVENTION OF NORTHERN OHIO.

*Object.* The object of this Convention shall be the promotion of Dental Science, fraternal intercourse, and the advancement of the general interests of the profession.

*Officers.* The officers of this Convention shall be a President, one Vice President, two Recording Secretaries, one Corresponding Secretary, and one Treasurer.

*Membership.* Any dentist may become a member of this Convention, by reporting his name to the acting Secretary.

*Finances.* Each member shall pay his proportion of the expenses incurred by the Convention.

*Business.* The business of this Convention shall be conducted according to parliamentary rules.

Rules of Debate. 1. Each member shall be limited to ten minutes, when he has the floor.

2. No member shall speak more than twice on the same question, unless permission be granted by the Convention.

Questions for Debate. 1. What are the causes of decay of the teeth?

2. What are the best means of preventing and arresting decay, and preserving the teeth?

3. What is the best treatment of exposed dental pulp?

4. What is the best treatment of alveolar abscess?

5. Is the porcelain mode of inserting artificial teeth, superior to other modes?

6. Is crystal gold superior to gold foil for filling teeth?

7. Miscellaneous Business.

The committee being mostly strangers to members, respectfully refer the nomination of officers to the Convention, recommending that they be elected by ballot.

All of which is respectfully submitted.

Thomas McCune,<br>C. S. Pleasants,<br>W. B. Ingersoll, } Committee.<br>C. H. Harroun,<br>Alfred Terry.

On motion of Dr. J. A. Robinson, the report was accepted, and, on further motion adopted. It was subsequently moved and carried, to set aside so much of the report as recommended the election of officers by ballot; and the election was conducted *viva voce,* and resulted as follows: President, F. S. Slosson; Vice President, B. T. Spelman; Recording Secretaries, L. C. Ingersoll, George Watt; Corresponding Secretary, W. H. Atkinson; Treasurer, B. F. Robinson.

It was moved and carried to elect a committee on finance.

Drs. Thos. McCune, S. P. Huntington and A. A. Harris were elected said committee.

On taking the chair, Dr. Slosson, the President elect, made a few highly interesting and appropriate remarks. He thanked the Convention for the honor conferred on him. He said his tongue was not "as the pen of a ready writer," but refused now to give utterance to his thoughts and feelings. He cordially thanked those in attendance for their presence, for the encouragement they thus gave the enterprise in which we are all engaged. He remarked that he was not familiar with parliamentary order, but trusted that the same liberal spirit which induced them to place him in his present position, would enable them to overlook any failures he might make in the discharge of the duties now devolving upon him.

On motion of Dr. W. H. Atkinson, it was resolved to proceed now to the discussion of the first question:

"*What arethe causes of decay of the teeth?*"

Dr. Watt, by request, opened the discussion, remarking that the question is important and direct. A knowledge of the cause being always necessary in arriving at correct principles of treatment, this is a fit question with which to open our discussions. In asking, "what are the *causes*," it is clearly implied that more than one agent is capable of producing dental decay; and it is also implied that there are different kinds of causes. He would confine his remarks to the immediate, or exciting causes, and remarked that these are to be looked for among the acids. This we might infer from the composition of the tooth. An active alkaline base, and a salt, which, with several acids, acts the part of a base, constituting its principal bulk, would lead us to look to the acids capable of uniting with these, as the principal, if not the only agents directly concerned in the production of dental caries. The teeth are not acted upon by all acids, nor do those capable of acting on them produce like results. The caries caused by nitric acid is very different from that which results from sulphuric or hydrochloric acid. Hence, dental caries is not a distinct disease, but is the result of chemical action; and its phenomena vary with the character of the agent introducing it.

Dr. J. A. Robinson inquired of Dr. W. what acid, injurious to the teeth, was generated in the system, from the use of sugar and saccharine substances generally.

Dr. Watt replied that a variety of acids might result, the particular one being determined by the modifying circumstances present. That the acid, derived from this source, most disastrous to the teeth, is the glucic, an acid remarkable for its affinity for lime.

Dr. W. H. Atkinson said that while it was true that where a tooth is sound and healthy, ordinary acid and alkaline solutions do not act on it, yet if the enamel be removed or checked by mechanical violence or otherwise, the agent then exerts its influence on the dentine, and we often find the gelatinous portion remaining, while the calcareous matter is totally dissolved and washed out. When the dentine is exposed, it sometimes appears to be carbonized. He did not know how this appearance was produced. The acid being confined, as in the case alluded to, is highly disastrous in its effects, and the decay often progresses with great rapidity. Cleanliness—the removal of acids as fast as formed—is the remedy. The usual positions of the decay, he thought, threw some light on the nature of the causes producing it. It was found in the depressions of the grinding surfaces, where the enamel is likely to be defective, and where decomposing substances are liable to accumulate: it is also found on the approximal and labial surfaces, occupying the latter position most frequently in the mouths of those who laugh but little. Mechanical attrition, accordingly, preserves the teeth by removing deleterious agents from their surfaces. The chewing of sticks, as practised by some, and even tobacco, may, in this way, act as a preservative. White decay, he believed, always resulted from nitric acid, or from alkalies.

Dr. Horton now suggested that the roll be called, and that members be requested to speak as called. The suggestion was adopted.

Dr. J. E. ATKINSON had not intended to say anything, and would merely remark that, as a general thing, acids, and acids only, cause dental decay.

Dr. W. B. INGERSOLL remarked that the question is constantly asked, "Why do every body's teeth decay?" It was difficult to answer. A man comes into our office with his teeth all sound; yet he never cleanses them—eats what he pleases—cracks nuts and other hard substances, and believes all this fashionable cleansing of teeth is a humbug. Now, what must the dentist say? We must look behind the immediate cause—back to the predisposing cause—back to the primary formation of the tooth.

Dr. HARROUN never allowed his name to be called without answering; but he did not expect to talk—came to listen and to learn, and, also, to represent the city in which he resided, which, he believed, had never been represented at any meeting of the dental profession.* He thought it should have a representative, and his patrons thought so too.

Dr. L. C. INGERSOLL said he was not going to apologize for unpreparedness; yet they must not on that account, infer that he was prepared. The public mind has various theories in regard to the decay of the teeth. One tells us, "I took so much medicine that my teeth all decayed;" another says, "My children eat so much sugar that their teeth are all destroyed; others, that alum, and kindred substances used in bread, have ruined their teeth. In general, it is safe to say that the immediate cause is the presence of acids; yet, in Surgery, as well as in Theology, there are sins of omission, as well as of commission. It is generally admitted that young persons do not grow up as healthy and vigorous as they once did, that there is a general failure in the American constitu-

* Dr. H. is under a mistake. His city was represented at the joint meeting of the "American Society" and "Mississippi Valley Association," in Cincinnati, by the late Dr. B. N. Freeman, who died during the meeting referred to.—EDS.

tion, an observable deterioration of the race. This deterioration manifests itself on every part of the constitution, and, of course, upon the teeth. Here, then, we have one reason of the decay of the teeth; and, in the universal neglect of these organs, we have another. In a healthy state of the constitution, when acids are generated, alkalies are already present to neutralize them.

Dr. HUNTINGTON said that, in addition to the action of acids, malformation of the teeth and mechanical violence are fruitful sources of decay. He never saw a person have good teeth long, who was in the habit of cracking nuts with them.

Dr. McCUNE wished to know, if acids and neglect be the cause of decay of the teeth, why Europeans have almost invariably good teeth. They, said he, never brush their teeth, and they use acid foods in great abundance. To find the leading cause of the decay of the teeth, we must look back to the infant—back farther still—to the parent. The climate and the mode of life have each their influence on the destruction of the dental organs. Times are much changed. The digestive functions were formerly good, the laws of life were less frequently violated, and the result was good constitutions. Now how is it? We eat, and *eat*, and EAT—bread, and pork, and beans, and cabbage—all that we can—then, pies and puddings, as if we had eaten nothing before them. The result is almost universal want of health. Look at the masses of our community. Look at ourselves, in this Convention. Who of us looks healthy? Well, we marry, and our women are as weakly as we, and more so. Can we, under such circumstances, expect healthy offspring? Can we expect a healthy development of any of the tissues? The vitality of the teeth is so low they have not the power of recuperation, as manifested in other parts of the system. The great predisposing cause of decay of the teeth is constitutional—the exciting cause is the action of acids.

Dr. WILLSON said that the remarks, thus far, had been principally confined to the chemical influences. He thought the mechanical should not be overlooked. A crowded denture usually indicates a tendency to decay. By mechanical causes the enamel is often fractured or abraded, then the acids have free access to the dentine, and produce their destructive effects. He thought that the presence of alkalies had a tendency to induce absorption or recession of the gums, thus exposing the necks of the teeth to the action of destructive agents. We frequently find teeth, and especially the wisdom teeth, decayed as soon as through the gum. He believed that mechanical action generally opened the way for the influence of chemical agents.

Dr. Moore said he never got into a place he could not talk but once. That time he attempted to extract one of his own teeth, got it partly out, and had to run a mile to have the operation completed by a surgeon. He must confess he was not talkative, by the way. He remarked that the question before us is important, and difficult to answer. That which destroyed one person's teeth often had no effect on those of another. Instances of this kind are seen in members of the same family—among those who eat, drink, and sleep alike. Some who eat pickles, use tobacco, and indulge in deleterious habits, generally have sound teeth, while others who give these organs the most careful attention, fail to preserve them. One thing seems to be demonstrated, that is, the teeth will decay, and no one can, in many instances, tell why.

Dr. Harris said that he agreed mainly with the remarks of Dr. McCune. He did not think the whole matter was constitutional. Different kinds of caries result from the action of different agents.

Dr. Horton remarked that mechanical causes expose the dentine to the action of destructive agents, but the chemical action of an acid was necessary to the development of caries. The decay commences at points where the acid is confined. To dissolve any substance readily, we pulverize it; hence, if mechanical abrasion precede, the chemical action is facilitated. One cause of the generation of acid in the system is the effect of climate. A chemist had suggested to him to examine the fluids of the mouth, in which the teeth were decayed, with test paper. He had done so, and had almost invariably found them acid. They should be neutral. The inhabitants of the Mississippi valley were prone to febrile diseases, which had a tendency to induce an acid state of the fluids of the mouth.

[A few concluding remarks on this question, by Drs. McCune, Robinson, Atkinson, and the President, are lost by an accident which befel our notes. As they occupied but a few minutes, it is hoped that the loss is not serious.—W.]

On motion of Dr. W. H. Atkinson, it was

*Resolved*, That we invite the medical and scientific men of the city to sit with us in the deliberations of this Convention.

On motion, adjourned to meet this evening at seven o'clock.

FIRST DAY—EVENING SESSION.

The Convention met according to adjournment. The committee on finance presented their report which was accepted and adopted. [The report is not found among the papers.—W.] The Convention then proceeded to the discussion of the second question:

"*What are the best means of preventing and arresting decay, and preserving the teeth?*"

Dr. Spelman said he was not in the habit of speaking, but he might as well deliver his maiden speech now as any time. He thought that more interest would be manifested on this question, than on any other one before us. In general, decay of the teeth might be prevented by cleanliness. He had found decay on the labial surface of the teeth, difficult to treat successfully. We are all agreed in regard to the importance of absolute cleanliness; but the question arises, how is it to be applied? Much good may be derived from the use of appropriate soaps and dentrifices. Mechanical means are to be relied on for the removal of foreign substances from the teeth. To arrest the decay, the tooth is to be properly filled. He felt a delicacy in giving his views on the proper mode of filling, when so many older practitioners were present.

Dr. Willson said the question is the prevention of caries,—we were proceeding to this before we had properly defined the cause. The means to be used are various. The proper regulation of the child's teeth was important; and so was cleanliness. If we could ascertain with certainty, the constitutional causes, then we could adopt more rational treatment; but now we are obliged to treat merely the symptoms. We can not change the laws of nature. But, he said, it would be presumptuous in him to try to enlighten those present on this subject.

Dr. McCune remarked that it had been assumed that cleanliness is the first great means of preventing the decay of the teeth. In his remarks on the preceding question he had maintained that the cause is constitutional. If so, the treatment must be addressed to the constitution. Instead of spending millions for the instruction of our children in music, dancing and painting, we should spend a part in teaching them the laws of life,—in teaching people that they can not violate the laws of health with impunity,—in teaching them that just a certain quantity of gastric juice, and a definite quantity of bile are secreted, and that they will accomplish the digestion of a corresponding quantity of food and no more. Physiology should be taught in all our schools, for sound, healthy teeth, are the result of sound and healthy constitutions. But we can not accomplish all this; and, therefore, decay will be found, and the question then is, how to arrest it. The answer is, fill the tooth properly.

Dr. J. A. Robinson didn't know that he could do better than to continue the remarks of Dr. McCune. He would, therefore, make a few remarks on the best mode of filling. To succeed properly, in this operation, he said that dentists must have the best of instruments, must take more time in its performance, must be honest, and well paid for their labor. Dentists did not always do the best they could for a tooth, because they could not afford it at the price they received. Let it be the determination of every one to do the best he can in every case, and charge an equivalent for his time and labor, and improvement will, at once, be manifest.

Dr. W. H. Atkinson remarked that the best way to prevent the falling of a house, is, to have every part of the foundation and walls of good material, and properly laid. To apply the figure, the mother's influence might represent the foundation, and the bony textures, the walls. Look at the children fed on city milk, and on those in the country who use the article in its purity. The decay of the teeth is to be prevented by living in accordance with physiological laws; but if these laws have been already violated, and it is now too late to prevent decay, we must do the best we can. Good material is better than bad, even on a bad foundation. Before closing he would make one suggestion in regard to filling, when the decay closely approximates the pulp. He used, with success, as a non-conductor, a horn cap, which he moistened with creosote before he applied it.

Dr. L. C. Ingersoll said, that in considering this question, he would not go back to notice the fact that all constitutions are defective. He supposed that, in spite of all the protests of medical men, in regard to the laws of life and hereditary descent, people will go on, as formerly, marrying according to their own fancy. He was, therefore, tired of the stale saying, "You were not made right," as it was of but little practical importance. He was surprised that people know so little in regard to the value of the teeth. Many become almost infidels, rising in rebellion against the wisdom of their Creator, because they regard the teeth as instruments of torture, rather than useful organs, necessary both to health and enjoyment of life. He narrated incidents showing the narrow, contracted views of the public in regard to dental surgery, and also referred to the case of a physician, who, in a sneering tone, remarked to a patient, "If you wish to save your teeth keep away from the dentists." Dentists must not only be enlightened themselves, but they must enlighten the people.

Dr. Dunn remarked that the subject was one in which he took great interest, but as he was not in the habit of expressing his views in public, he presumed he could add but little that would be of interest. He concurred in Dr. Ingersoll's remarks in regard to enlightening the people. It was evident that nearly all who apply to the dentist do so only when impelled by physical pain. He would like to hear the different modes of treating irregularity. He regarded this as a prominent cause of decay, and, of course, its correction would be an important preventive.

Dr. Harroun remarked that he could think, but could not always give expression to his thoughts. He saw cases every day where the patients had neglected their teeth till they became painful, and then came to have them extracted. He never extracted temporary teeth if he could avoid it. He regarded premature extraction as a leading cause of contraction, irregularity, and, consequently, caries. After decay has commenced it is arrested with certainty, only by filling.

Dr. W. B. Ingersoll would take it for granted that ordinary cleanliness had been resorted to. Still some things had not yet been noticed. He had observed that cylindrical teeth are less liable to decay than those differently shaped. When there was a crowded condition of the teeth, he sometimes relieved it by filing, if caries had commenced on any of the approximal surfaces.

Dr. J. E. Atkinson remarked that the best way to *prevent* decay of the teeth is to live right; to *arrest* it, fill properly. All he could say on the subject would amount simply to this.

Dr. Strickland remarked that defective constitutions, and filling teeth had been already noticed. To avoid going over ground already occupied, he would take a common class of cases. Persons from fifteen to twenty-five years of age, with tolerably good teeth, none decayed, may wish to have their teeth examined, and to be advised in regard to the care of them. Now, what advice shall, we give them? Can we insure to this person a permanently sound and healty denture? As already suggested, he would advise cleanliness; but the question arises, by what means is this to be attained? The brush is a popular appliance, and it is good; but it is not all that is wanted. People must be taught, in the language of Dr. Parmly, that there are *four* sides to a tooth. The brush is inapplicable to the approximal surfaces; and, to cleanse them, he recommended floss silk, or thin tape, with the addition of chalk, or a kindred substance, when necessary. The indentations should be carefully watched, and incipient decay promptly treated. The same class of persons may come to us, a little later in life, with teeth decayed. The cavities are found in the depressions of the grinding surfaces, on the approximal, and on the labial surfaces. Now, what is to be done? The decay is to be arrested by filling, or by removal with the file. Inquiry has been made in regard to the manner of polishing filed surfaces. I would polish them —somehow—soft wood and pumice, followed with chalk may answer. He would take as much pains in polishing a filed tooth, as in polishing his finest fillings.

Dr. Watt remarked that, in the prevention of decay, our means are used with the greatest advantage in the earliest periods of existence. With reference to this, he frequently prescribed for patients who, not only had no teeth, but whose tooth-germs were only in process of formation. If it be true that the teeth of our children are deficient in phosphate of lime, nothing seemed to him so rational as to supply this ingredient. To be received in time to give due aid in the developement of the deciduous organs, it is necessary that it be supplied through the mother. He was in the habit of prescribing it to mothers whose children have defective teeth, during the latter months of gestation, and the earlier periods of lactation, and he generally received their most hearty thanks for the prescription. The phosphate may often be administered directly to the child, with decidedly beneficial results. It is always indicated when the constitution gives evidence of a deficiency of ossific matter. On the second part of the question, arresting decay, he would say nothing at present.

Dr. Fisk inquired in regard to what is called "chemical food," which he said was a solution of phosphates, and was not a nostrum.

Dr. Slosson remarked that a loosened state of the teeth, with spongy disease of the gums had not yet been noticed. A physician had called his attention to a case of neuralgia, with the mouth in the condition described, loosened roots of teeth remaining in the gums. He told the physician that a dentist would, perhaps, be of as much use as a doctor. He cleansed the teeth and treated the gums, and the neuralgia was relieved. He spoke of a similar case. A married lady had periodical neuralgia, applied to a physician, who prescribed blue pills. He saw her in two weeks, the teeth loose, and the gums spongy and inflamed. With treatment like that of the preceding case the recovery was complete.

Dr. Bailey said that he had intended to make a few remarks, but he had been mainly anticipated by Dr. Strickland. He advised his patients to use the brush and floss silk, immediately after meals,—if used but once a day—after tea. In regard to the arrest of decay, by filling, he had not time to give his views now.

Dr. Horton remarked, that if we had a reliable theory of the cause of decay, we might have a correct one in regard to the remedy. This morning we had a chemical theory, explaining the cause of the decay of the teeth ; now, to preserve them, we must have a counteracting chemical influence. He reported a case in which the deciduous molars were crowded by the permanent, and the result was tooth-ache. He separated the deciduous molars, which were already imperfect, and the pain was relieved. If the fluids of the mouth exhibit an acid reaction, he recommended the use of alkaline washes, as tooth-soap, or something still more alkaline. As to the ignorance of the physician referred to by Dr. Ingersoll, he remarked, that physicians look upon medicine as so far above dentistry, that they consider the latter of but little account. Many who call themselves physicians, are very ignorant of the principles of dental surgery. He referred to a case in which several physicians, in giving evidence, failed to remember the number of the deciduous teeth.

It was, on motion, *Resolved*, that we close the discussion of the second question, with the present session, and the discussion of all the questions in the report, by to-morrow noon; and, also, that the call of the roll be dispensed with.

On motion, adjourned to meet at 8 o'clock to-morrow morning.

SECOND DAY—MORNING SESSION.

The Convention met a little after the appointed hour,—was called to order by the President, who announced, as the first business in order, the discussion of the third question,

"*What is the best treatment of exposed dental pulp?*"

Dr. J. A. Robinson remarked, that, as there seemed to be a backwardness in commencing, he would offer a few suggestions. He would discuss the question with reference to the six front teeth. In operating on these, he remarked, the pulp is often suddenly and unexpectedly exposed. He said the treatment was simple, and there need be no dispute about it. He applied chloride of zinc to cause a slough,—then followed with creosote. He believed 99 per cent. of such teeth can be saved by this treatment. He at one time used caustic potash as a "darling remedy." Its use in such cases was suggested to him by the late venerable Dr. Flagg, of Boston. It cauterizes the nerve, or, in the language of Dr. F., makes soap of it. He had abandoned the potash, and now used chloride of zinc, which is milder. Dr. Palmer (?) of Warren had recommended a reliance on creosote and pressure, in the management of such cases. He introduced a pledget moistened with creosote, and filled over pledget. He, (Dr. R.), recently saw a case which he treated in this way, which is doing well. Dr. P., however, states that he saves only about

40 per cent. of cases treated in this way. He, (Dr. R.) relies on chloride of zinc as an escharotic, and on creosote, as an antiseptic, to heal. But if soreness had intervened, he used arsenious acid. In reply to a question, he stated that he could save *all* the molar teeth treated in this way. He filled the fangs, and postponed, for a time, the filling of the cavity of decay.

Dr. Strickland remarked that his treatment had differed entirely from that spoken of in reference to Dr. Palmer. He endorsed the destruction of the pulp, and filling the fangs. He had filled incisors in this way, eighteen years ago, which were still preserved. This treatment, in his hands, had been more successful with the front teeth than with the molars. He would like to know if the pulps treated by pressure, etc., are still alive. Is the success merely that the teeth still remain in the mouth? A good constitution, said he, may be able to absorb all the results of a decomposing pulp. He did not believe one half the cases treated as described would be successful. We must have the facts.

Dr. J. A. Robinson responded that in treating exposed pulps in front teeth, he never used arsenious acid, but chloride of zinc and creosote, and that the pulps did not die, nor did abscesses form. He spoke positively, from twenty years' experience.

Dr. Strickland did not believe that arsenious acid always changed the color of the tooth. If applied in proper quantity, and not left too long in the cavity, it was not likely to do so.

Dr. B. F. Robinson could not say that he had been as successful as his brother had reported himself. He had teeth in his own mouth which had been treated with chloride of zinc and creosote, in which he knew the pulps had died. He endorsed all that had been said by Drs. R. and S. in regard to the filling of fangs. He had tried almost every mode suggested. He thought he had saved some treated with caustic potash, and also, with chloride of zinc.

Dr. Strickland remarked that he obtained his first ideas on the use of arsenic, from a physician at Cuyahoga Falls, who used a mixture of arsenious acid and sulphur for the destruction of cancerous tumors. This was before the article was in use by the dental profession.

Dr. W. B. Ingersoll remarked that he had some experience in the treatment of exposed pulps. He did not like to leave the arsenic in the cavity longer than from six to twelve hours. If the pulp was sensitive but still healthy, he used creosote and a non-conductor, and filled over it. Was not certain that he saved alive more than half the pulps thus treated. Abscesses frequently formed. He had resorted to Hullihen's operation, a few times, successfully. He would inquire of Dr. Strickland if, when the pulp was covered by softened, or decomposed dentine, but still healthy, he would extirpate it and fill the fangs.

Dr. Strickland replied that he certainly would, if the bone was decomposed. It would prove a source of irritation if left.

Dr. W. H. Atkinson remarked that no one ought to be so selfish as to wish to advertise himself in such a meeting as this. As to the discoloration of the teeth, he maintained that it resulted from a disorganization of the blood globules. He remarked that we had all capped and filled, and had seen abscess follow. When the pulp is exposed, but healthy, it may be saved in a vast majority of cases. But if suppuration, or ulceration on the surface of the pulp has taken place, if we fill without treatment, we lose the tooth. The point to be determined is, is the pulp entirely free from inflammation ? As to extirpation and fang filling, he remarked, that when arsenic exerts a deleterious influence, it does so by injuring the periosteum. He would leave the arsenic in the cavity from two to six hours. He prefers, when he has the control of the patient, to use creosote. By repeated applications of this, the pulp would lose its sensibility till it could be removed readily with an instrument. The time usually required was from four to six weeks. Whether it is better to use arsenic or creosote is a question of time. He had seen cases in which he had filled over exposed pulps, with tin, in the mouths of some school-boys, and afterward, on removing these to fill with gold, he had seen the pulp covered with cementum. He believed that our ability to save exposed pulps is inversely as the age of the patients. He protested against destroying all exposed pulps. In his management of exposed pulps he might almost say that he used creosote first, last and all the time. It neutralizes deleterious agents. In applying it, he used a pledget on a blunt instrument, and applied pressure sufficient to bring the creosote in contact with the entire exposed surface. He believed that the coagulum formed by the action of the creosote on the surface of the pulp was the matrix in which the new layer of cementum was deposited. He believed it was not always practicable to reach the point of the fangs of the molars. He inserted a small gold wire as far up as practicable, and filled around it. He described his mode of making " vent fillings" with which, he said, he had been very successful. [We failed to get Dr. A's remarks on this point. Will he write us his mode, for the next issue of the Register ?—W.]

Dr. Willis would inquire if the coagulum caused by the creosote is not dead ; and, if so, how does nature dispose of it ? Exposed pulps almost always give him trouble. The bone was the natural covering ; and others could not be perfectly adapted. How is the dead portion of nerve got rid of, he regarded as worthy of consideration.

Dr. Watt said, that when the exposed pulp was in a healthy state, and he intended to fill over it, he preferred the action of tannin to that of creosote on its surface. It formed a similar coagulum, but a much thinner layer, and one more thoroughly insoluble. In other words, he regarded tannin as a more perfect local antiseptic than creosote. If the pulp be inflamed he used cresote and filled over it with gutta percha, giving nature time to dispose of the slough before filling with gold. The layer of coagulum formed by the creosote was not "coagulated lymph" nor "coagulable lymph," but a compound of creosote and albumen, and, consequently a dead substance. He thought that the cementum, or osteo-dentine, referred to by Dr. A., was not deposited in this, but beneath it. Where the pulp is healthy, and still covered by a layer of the gelatinous portion of the dentine, he would not extirpate it, nor, would he consider it, when strictly speaking, a case of exposed pulp. This portion of gelatine, by proper treatment, forms a perfectly adapted, non-conducting cap. He removed the decayed matter from other parts of the cavity, then inserted a concentrated solution of tannin, and filled over it with gutta percha. His object was to convert the "softened dentine" into *leather*, which is only tannate of gelatine. He afterwards removed the gutta percha and filled with gold.

Dr. Strickland remarked, in explanation, that when he had control of the patient's time, he frequently pursued similar treatment; but when he had not, and it became necessary to proceed at once, he extirpated the nerves and filled the fangs.

Dr. L. C. Ingersoll said that he had often felt at a loss in regard to the treatment of teeth where the pulp was protected only by the gelatinous portion of the dentine. After reading an article in the Dental Register, by Dr. Watt, on "The Action of Topical Remedies," he had experimented with tannin and a solution of gelatin, and found that the result was a firm, elastic, insoluble substance. He argued that, by converting the gelatin which covers the pulp into a like substance, we have afforded it the best possible covering under the circumstances. He regarded it as an interesting fact that tannic acid forms insoluble compounds with the constituents of living tissue. Its absorption was thus rendered impossible, otherwise it would form a most active poison. He was satisfied that osseous matter was sometimes deposited over pulps thus treated.

Dr. McCune remarked that the objects for which we were assembled, were beginning to be brought out. We had been told that under certain treatment, 99 per cent. of cases were successful. He feared that the cases were not followed up with sufficient care, to observe the unsuccessful cases. He remarked that nerves were exposed while excavating, and, frequently filled without treatment, and many operators he presumed, flattered themselves that the teeth were saved. In general, he believed, such cases resulted in suppuration and abscess within two months. He said he had not been very successful in treating exposed pulps. The nerves would die. He hoped they would not tell it, but he must acknowledge it. If he had confidence in the treatment recommended by Drs. W., A., and others, he would try it; but he had not. And the *pressure system* would not go down with him. He believed in always telling the patient the real condition of the tooth, and always expressed a doubt about saving it if the pulp was exposed. He generally extirpates exposed nerves, but has sometimes saved them without.

Dr. Spelman said that Dr. Palmer's (?) name had been used in connection with pressure on the pulp. Dr. P. did not intend to retain the pulp under pressure; and the pledget of linen left in the cavity was intended to absorb any matter thrown out by the nerve while healing. The linen was put in dry. He had filled over exposed nerves, after treatment, but he did not know how successful he had been. His patients might have become disgusted, on account of pain, and gone to another dentist to have their teeth extracted. It was not safe to infer that our treatment is successful because the patient does not return.

Dr. J. A. Robinson protested against the use of arsenic in the front teeth. He did not see that any good could be derived from the use of tannin. He used chloride of zinc and creosote as escharotics. He would as soon rely on caustic potash as the chloride of zinc; but the latter caused less pain. He had cut off and cauterized the nerve with success.

Dr. B. F. Robinson feared that many of the successful cases of exposed pulp were like the young surgeon's case of amputation: He regretted that his friend had not witnessed his operation,—it was so beautiful, and such a complete success. The friend regretted his absence, but was much pleased to hear of such satisfactory results, and inquired how the patient was now doing. "The patient!" said the surgeon, "why he died!"

Dr. Harroun said that he had treated exposed pulps in various ways. He prefers removing the pulp by mechanical means, where practicable, rather than destroying it with arsenic. He narrated, in illustration of his mode of practice, two cases treated successfully. If the pulp was but slightly exposed, he cauterized it with a combination of nitrate of silver and creosote, and filled, using a non-conductor and gold cap over it; and was generally successful in such cases.

Dr. Willis alluded to the great practical importance of this question, and remarked that he could not report himself as successful as some appeared to be. When the nerve is exposed, he treats with creosote, and filled the cavity with wax, and awaits the result. He had failed oftener than he had succeeded, and believed that an operation which brought a foreign substance in contact with a living pulp, was likely to fail.

Dr. Barrett said that to destroy the vitality of the pulps in molar teeth, he used arsenic. In the front teeth he removed the nerve with a fine gold wire, and filled the cavity without any previous application. He had endeavored to save exposed pulps by the use of caps and non-conductors, and had been satisfactorily successful; but, if the pulp was inflamed, he had sometimes failed. He had used tannin and kindred agents, on softened dentine, to avoid exposing the pulp, and had been successful. From suggestions heard to-day, he would experiment further in this direction.

Dr. Willson remarked that his experience accorded very nearly with that of Dr. J. A. Robinson. He succeeded in nearly 99 per cent. of his cases. To exposed pulps he applied a combination of morphine, quinine and creosote,—then removed from the cavity all softened matter. Where there was slight pain, it might be allayed by tincture of galls. He would leave to others, to discuss pathological conditions.

Dr. Bailey applied creosote and morphine, from three to six hours, and washed out the cavity with tincture of rhatany, or a similar astringent, before filling. In filling, he left a space between the cap and the pulp, to allow the latter to expand when inflamed.

It being now nearly the hour fixed for the close of the regular discussions on the questions contained in the report, it was agreed to take up the sixth one, leaving the others to be brought up, if desirable, under the head of "Miscellaneous Business."

"*Is crystal gold superior to gold foil for filling teeth?*"

Dr. W. H. Atkinson remarked that the subject of crystal gold was not generally understood by the profession, inasmuch as but few had used it to the extent necessary to a knowledge of its properties and uses. Except in small cavities and in fangs, he could fill better with crystal gold than with foil. Crystal gold, said he, packs only directly under the instrument, the points of which should be sharp. The gold should be carefully packed against the walls of the cavity, and then the middle portion can be filled with rapidity. The gold should be torn apart, with fine pointed instruments, without bruising, or condensing it. It should be packed evenly in the cavity, being careful to go but once over the surface of each pellet, with a serrated instrument; for, if twice gone over, the sharp projections, formed on the surface of the gold by the first application of the instrument, will be broken off, and the succeeding pieces will not adhere so readily.

Dr. B. F. Robinson said that he had not used crystal gold enough to satisfy him that he could do better with it than with foil.

Dr. Watt remarked that he considered the relative merits of the two forms of gold, a question of less practical importance now, than formerly. Crystal gold may be set down as a fixed fact; but it would never entirely take the place of foil. It is unprofitable to discuss the question of which is the better, while both are good. He would not like to be without either one of them, and often used both in the same cavity. He thought it probable that all could be done with foil that could be done with the crystals; but, in building up, to restore the contour of the tooth, the latter could be used with far greater facility; and he would greatly prefer it in frail teeth.

It being now 12 o'clock, the Convention adjourned to meet at 2 o'clock, P. M.

SECOND DAY—AFTERNOON SESSION.

The Convention met according to adjournment. The President announced that "Miscellaneous Business" was now in order.

Dr. McCune offered the following resolutions, which, after being enforced by appropriate and pertinent remarks from several members, were adopted, seriatim:

*Resolved*, That each member of the Convention be requested to keep a strict record of all the nerves treated by him, and examine them, so far as practicable, before coming to the next meeting, and report his success at that time.

*Resolved*, That this Convention appreciate the efforts of our Dental Colleges, to elevate the standard of the dental profession, and to qualify young men for sustaining that standard.

*Resolved*, That this Convention condemn the practice, pursued by some, of puffing themselves into business, by flaming advertisements and circulars, proclaiming that they have instruments unequallad, that all their operations are infallible, as they can prove by Dr. A. and Governor B., and trying to convey the idea that they are the great centre and circumference of all dental excellence; as savoring of quackery, and entirely unworthy of a liberal profession.

*Resolved*, That we are happy to recognize Prof. Watt of Cincinnati, as a member of this Convention, and shall always be happy to see dentists from abroad, and would cordially invite all to participate with us in our discussions.

Dr. L. C. Ingersoll offered the following, which was adopted:

*Resolved*, That, in the most emphatic terms, we discountenance the preparation of students for the practice of dentistry in a few months, and recommend most strongly, a thorough education in a Dental College.

Dr. W. B. Ingersoll described a modification of molar forceps which he had found advantageous.

Dr. C. F. Ingersoll remarked that he had with him an instrument constructed on the plan described by his brother. He exhibited the instrument, and pointed out what he considered advantageous in regard to it. [The peculiarities are difficult to describe on paper, and we will, therefore, not make the attempt.—W.]

Dr. L. C. Ingersoll described an experiment which he had tried with an amalgam filling, in an extracted and thoroughly dried tooth. The filling was made from a compound of silver and tin, with mercury. It contracted so much that a perceptible space was left, between it and the walls of the cavity. Now, he wished to know if, in certain proportions, a compound of these metals contracts, while in other proportions it expands, in hardening. He had positive evidence of contraction. Others maintained a corresponding expansion. He desired information on this point.

Dr. W. H. Atkinson remarked that it could be determined only by experiment. He referred to instances both of expansion and contraction resulting from crystallization. Dr. A. also made some remarks on alveolar abscess. He thought it probable that a majority of the profession still discarded all treatment of alveolar abscess. He spoke of teeth loosened by salivation, with pus discharged from around them, and inquired whether, in this condition, the pulp could be still alive. He maintained that it could.

Dr. L. C. Ingersoll inquired if Dr. A. considered this true abscess.

Dr. Atkinson maintained that the pathological conditions were the same as in true abscess. He said that the first step in the formation of abscess is the evolution of a minute quantity of gas. The case may still terminate in resolution. If it do not, the irritation arising from the presence of dead tissue results in the throwing out of coagulable lymph, and the inflammation terminates in suppuration. In perfect cure of abscess, he said, that almost an osseous union of the fang and bottom of the socket took place. In treating abscess, he sometimes cut through the process, to destroy the sac. He gave an interesting case of abscess, from the central incisors, successfully treated, and remarked that he was then bleaching the teeth with chloride of zinc. He reported a number of cases treated with similar success. He recommended, in some cases, cutting through the process, and dividing the periosteum to relieve congestion. If he found a calcareous body at the root, he broke it up with an excavator.

[Dr. A. was heard at considerable length, on this subject; but his rapidity of expression renders it impossible for an unpracticed reporter to do him justice.—W.]

Dr. Slosson spoke of a case of abscess, from the fang of a central incisor, which occurred some years ago. The tooth was elongated, and the gum swollen, with formation of pus, from one canine to the other. With a probe, he found an orifice through the process, and a roughened calcareous deposit around it. He removed the deposit, and treated the case with entire success,—there being still no return of the abscess. He referred to a case in his own mouth, the particulars of which we failed to obtain.

Dr. W. B. Ingersoll spoke of fangs which, when left open, gave no uneasiness, but were painful when filled.

Dr. W. H. Atkinson said that a gas was formed by the decomposition of dead matter in the fang, which escaped, when the fang was left open, but was retained, if it was filled.

The Convention then took up the subject of

ARTIFICIAL WORK.

Dr. Dunn remarked that he adopted porcelain work, in his practice, from necessity. His patients demanded, and would have something cleanly. Now, he was forced to say that each day's experience rendered him more and more in favor of the porcelain work, as manufactured according to Loomis' patent. He thought the entire profession would like it, if they would only give it a fair trial. He said those who had lost their teeth, had already suffered as much from decomposing materials in the mouth as they ought to. This work, he said, was absolutely cleanly; and could be perfectly adapted, so as to preserve the natural shape of the mouth and features, better than with gold, or any other style of work. It would masticate anything—was as strong as any other kind of work, when properly made. He reported cases in which, at the wish of the patients, he had removed gold jobs, and substituted this, giving to the patients the highest degree of satisfaction. To a question of the President, he said he did not know how many sets he had inserted. To questions of Dr. Watt, said that it was as well adapted to lower, as to upper dentures; and, that partial sets were not in his province—they belong to Dr. Wright. To a question of Dr. Strickland, said he did not attempt to make the work fit the rugae, however prominent they may be.

Dr. Spelman remarked that he might say something against porcelain work ; but it was evident that it was yet in its infancy. He said that the most accurate impression we can obtain is imperfect, hence, we have to use the pliers to correct the plate, in many cases, especially in the front part of the mouth. He said if we could obtain a philosophical fit, our plates would be held in place by a pressure of almost fifteen pounds to the inch. The nearer we approximated a philosophical fit, the more firmly would our plates adhere. If he were convinced that we could closely approximate a philosophical fit with porcelain work, he would advocate it. The work was very liable to break, and it was not practicable to repair it. He would like to know how to obtain a philosophical fit with this work.

Dr Dunn maintained that a more perfect fit may be obtained with this work, than with metallic plates. The method was, to take the model, and grind the work to fit it. He could not describe the grinding process so as to be understood; but he would be much pleased to demonstrate the method to any member, at his office. He could show, by his models, that he obtained perfect fits.

Dr. W. H. Atkinson inquired if he could calculate the shrinkage of his body, or, had he a body that did not shrink.

Dr. Dunn replied he could calculate the shrinkage.

Dr. Spelman remarked that just in proportion as porcelain shrinks but little, it becomes worthless. It was covered only by an enamel that fused at a low temperature; and when this was removed, the base was found to be so porous that all the fluids of the mouth would permeate it.

Dr. W. H. Atkinson wished that we knew more about porcelain. He inquired if the clay, in the body, was not the cause of the shrinkage, and whether it imparted any strength to the porcelain. He believed it did not. It was well known that the same porcelain shrinks more or less, according to the temperature to which it is subjected. He would, therefore, inquire how, in the manufacture of this work, a uniform temperature is to be obtained. Does Dr. Dunn use a pyrometer?

Dr. Dunn replied that he did not.

Dr. J. A. Robinson remarked that he had considerable experience in porcelain work; but he had not been very successful. He would not wish his worst enemy a severer h—l than to have to re-make two hundred jobs. He remarked that Satterthwaite's work would fit, and that Loomis' could be ground to fit sufficiently accurate to stay in the mouth. He exhibited a piece which he said it had not taken him over *three days* to grind. There was something, he said, about porcelain work, which induced it to stick when the fit was not very accurate. It would stick when a gold plate would not. He narrated an anecdote in confirmation of this: His son had a "specimen job" of porcelain work, made without reference to the mouth of any one. When on a visit to his son's office, an old lady looked at the piece,—admired it,—placed it in her mouth, and exclaimed, "Why it just fits me." There it stuck—was not displaced by the movements of the mouth in conversation, though it fitted but a little better than a horse-shoe would have done. He spoke of a case in which a piece of work was worn, successively, by three different persons. He said that people clamored for porcelain work, and he had to make it; but he was thankful that he made less and less of it. He denied that Loomis' work could be made to fit. Satterthwaite's would fit, but was not strong enough.

Dr. Moore said he did not like porcelain for partial sets; but he could fit the work to the mouth in much less time than that spoken of by Dr. Robinson. He considered good porcelain work the best extant, but porcelain often looked well when it was brittle.

On motion of Dr. W. H. Atkinson, it was

*Resolved*, That it is regarded by this Convention as derogatory to the standing of any dentist to charge less than one dollar each, for the smallest class of gold fillings, in Northern Ohio, exclusive of the cities of Cleveland and Toledo, where the fee for the same class should be one dollar and a half, per filling.

With the Vice President in the chair, Dr. Slosson offered the following resolution which was unanimously adopted:

*Resolved*, That we regard, as of great importance, the establishment of such a dental depot in this city, as shall meet the wants of the profession in Northern Ohio, and vicinity; and that we will use our best efforts to secure and sustain such a depot.

The Convention proceeded to determine the time and place of the next meeting; and it was resolved to meet again in Cleveland, on the first Tuesday of May next.

On motion, adjourned to meet this evening, at 7 o'clock.

SECOND DAY—EVENING SESSION.

The Convention met at the appointed hour. Dr. Spelman, Vice President, in the chair. The discussion of artificial dentures was resumed.

[We regret that we can not report the discussions of this session. They had progressed to some extent, before our return from tea; and knowing that we would be compelled to take the cars before the hour of adjournment, we made no notes. After a night's ride in the cars, two days' writing, and another night's ride before us, we were not "in the mood." We will only remark, that though our energies *evaporated*, there was no "giving down" in the Convention. The last hour we were with it, was its liveliest and best.—W.]

The evening being now far spent, Dr. McCune moved to adjourn, and said:

*Mr. President and brethren of the Convention*:—The question would naturally arise at this moment, have we accomplished the object for which this Convention was called? Have we done anything towards elevating the standard of our profession? (Many voices, "Yes.") And so do my own feelings echo, Yes. Well what have we done? We have discussed the various topics touching the profession. We have each thrown in our mite into the great reservoir of knowledge; and we have each been to imbibe at its fountain. I have been reminded, during our discussions, of a certain old farmer in Massachusetts, whose external appearance would indicate that he was not very bright, but his inner man told a different story. He was one day fixing a pig-trough through his fence, and fastening it down with a forked stick, when a gentleman riding along stopped his horse, and, after looking at the farmer a few moments, remarked, "Well, every fool knows something." "Yes," replied the other, "and every fool may learn something." (Applause.) Well, sir, if we have been anabled to learn something, we have taken one step towards elevating the standard.

Again, we have erected a platform, and proclaimed to the world, that our profession is based upon a scientific knowledge of its principles; that to be a good dentist, we recognize a certain curriculum of study, or a regular dental education as necessary. (Applause.)

We have been a set of jealous men,—that is, jealous of each other; and I do not know but we shall have to plead guilty to the charge. Have not a great many dentists shown a disposition to keep their knowledge and modes of manipulation to themselves, lest others should become wise, and be enabled to work as well as they, and thus intercept their practice? Let all such little, contemptible feelings be thrown overboard; and let us awake to the fact that the more *good* work there is done, the more confidence will the people have in dentistry, and the more work will there be for all of us to do. Thus we shall elevate our standard. (Applause.)

Again, we have shown to the world, by our resolutions, that our faces are set against that hydra-headed monster, quackery, that stalks abroad through the land, imposing morally, physically, pecuniarily and ignominiously upon our fellow men. (Applause.) Have we not resolved in our own minds that the great and paramount object of our labors shall be to confer the greatest amount of benefit upon our patients? And that, when a patient places himself or herself in our hands, we are bound by our honor to give them such advice as we think is for their best good, without any regard to our own pecuniary interest? (Applause.)

If this Convention has been instrumental in bringing any of us to such a determination as this, then have we done much to elevate the standard. I think I may safely say that our profession stands higher in our own estimation at least; and I am satisfied that it will stand higher in the estimation of the community when they shall know our principles.

Have we formed a society? Yes,—upon the broad democratic platform, that every man may come and contribute to our reservoir, and imbibe at our fountain.

Have we done any thing to promote fraternal intercourse? It seems to me, Mr. President, that my heart has grown warmer and larger every moment, since we came together; (Applause) and I am sorry that the time has come when it is necessary to make the motion that is now before the house; and that we are now about to exchange the parting friendly grip. Mr. President, I need not ask if these are the feelings of the other members of the Convention; for I can read it in their very countenances. (Applause.) I feel, sir, that each of us has now an acquaintance and sympathizing brother in almost every town in Northern Ohio; nor are they confined to Northern Ohio; and I will further presume that when any of us shall visit our professional brethren at their homes, we shall not meet with a cold-hearted, jealous surmise of, "what are you hear for ?" but we shall meet with a cordial and hearty welcome. (Applause.)

Gentlemen, may that fraternal feeling and confidence which has inspired us at this meeting, never grow less; and may we all be spared to renew our congratulations on the first Tuesday in May next. (Loud applause.)

The meeting then adjourned with three cheers for the success of our first Convention.

[And now, reader, you have the result of our first attempt at "reporting for the press." We have endeavored to be faithful, but we have made some mistakes. One very flagrant one we will tell you how to correct. We omitted to record the "applause," "laughter," etc. Well, you can imagine it in. Where any thing tolerably pointed is said, just think—"Applause;" if any thing sharp, "Loud Applause;" if amusing, "Laughter;" if witty, "Laughter and Applause." And you need have no caution in regard to overdoing it; for every member's heart seemed to be overflowing with good feeling; and it was altogether the most heartily cheerful and *cheered* meeting of the profession we ever attended.—W.]

